# Supplementary material for: High-salt diet inhibits tumour growth in mice via regulating myeloid-derived suppressor cell differentiation
Source: Nat Commun. 2020 Apr 7;11:1732. doi: 10.1038/s41467-020-15524-1 (PMC7138858; doi:10.1038/s41467-020-15524-1)
Supplement: Supplementary file 1 — Supplementary Information [file 41467_2020_15524_MOESM1_ESM.pdf]

High-salt diet inhibits tumour growth in mice via regulating myeloid-derived suppressor cell differentiation

He et al.

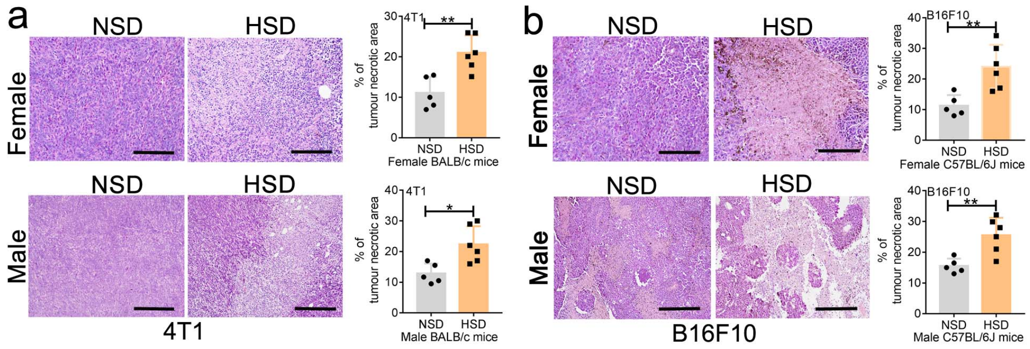

Supplementary Figure 1. HSD led to large necrotic areas in the tumour tissues. 4T1 or B16F10 tumour cells were subcutaneously injected into NSD- or HSD-fed mice until tumour tissues were harvested. Tumour sections from NSD- and HSD-fed mice with 4T1 (a) and B16F10 (b) cells were tested by H&E staining, and the percentage of tumour necrotic area in high-power optic (×200 magnification) field in stained sections (five fields for each section; n=5 individual tumours in NSD group and n=6 individual tumours in HSD group) was analysed. Scale bar, 100 µm. The two-tailed Wilcoxon rank-sum tests; n = 5 for NSD group and 6 mice for HSD group; $*p<0.05$ and $**p< 0.01$ vs. the NSD group. Bars are expressed as the mean ± SEM. n= 5 data points for NSD group and 6 data points for HSD group (the average number of five fields for each section). These experiments were repeated twice and were replicated with similar results. Source data are provided as a Source Data file.

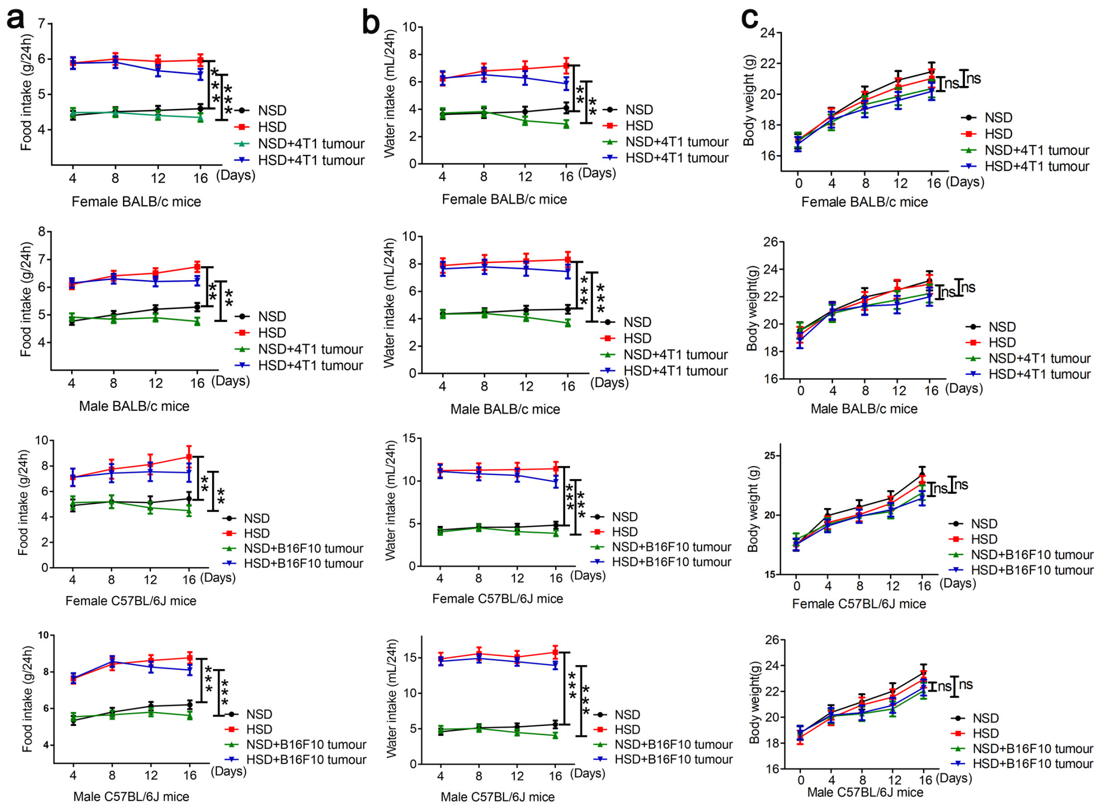

Supplementary Figure 2. The effect of HSD on food and water intake and weight of tumour-bearing mice. (a) The effect of HSD on food intake of mice. (b) The effect of HSD on water intake of mice. (c) The effect of HSD on body weight of mice. For all panels, the two-tailed Wilcoxon rank-sum tests; n = 10 mice per group; ns, not significant, ***p*<0.01 and ****p*<0.001 vs. the NSD group. Data are presented as dot plots extending to minimum and maximum values in one independent experiment and bar are presented as the mean ± SEM of 10 individual mice. These experiments were repeated twice. Source data are provided as a Source Data file.

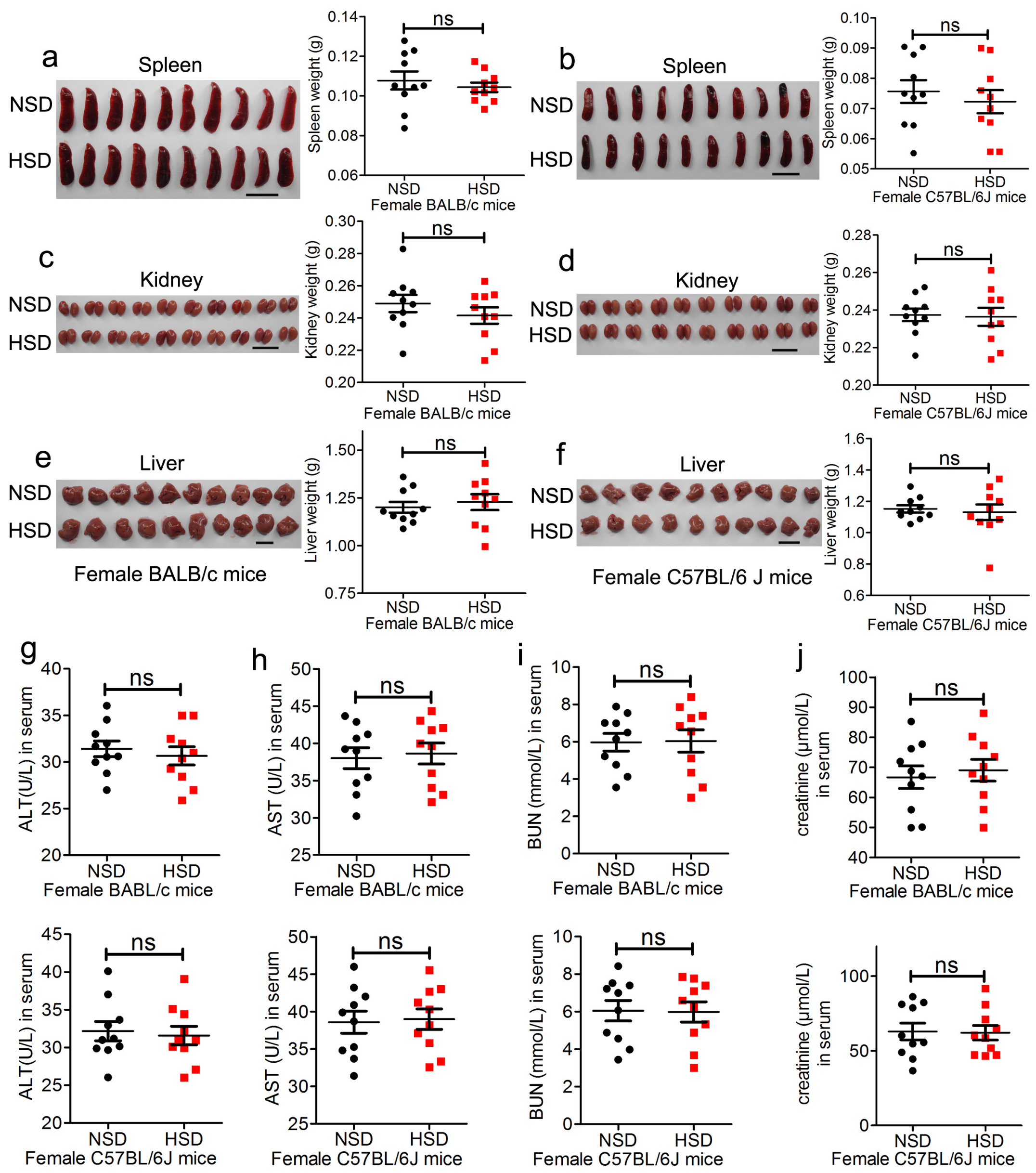

Supplementary Figure 3. The effect of high-salt intake on the health of normal mice. (a-f) Images of spleen, kidney and liver harvested from mice and their size. Scale bar, 1 cm. (g-j) The effect of HSD on the levels of ALT, AST, BUN and creatinine in both the NSD and HSD group. For all panels, the two-tailed Wilcoxon rank-sum tests; n = 10 mice per group; ns, not significant. Data are presented as dot plots extending to minimum and maximum values in one independent experiments and bar are presented as the mean ± SEM of 10 individual mice; each dot represents one mouse. These experiments were repeated twice. Source data are provided as a Source Data file.

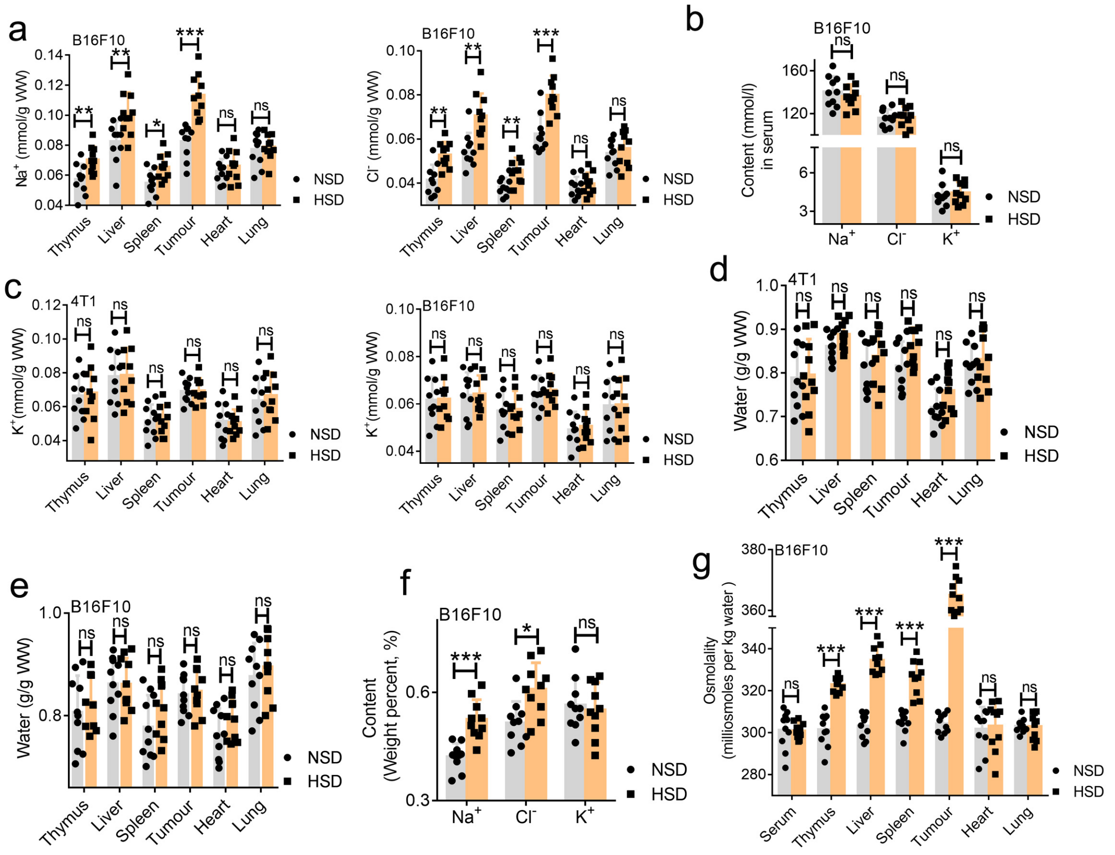

Supplementary Figure 4. The effect of high salt on salt storage in different organs. (a-c) Na^{+}, K^{+} and Cl^{-} content in different organs compared with plasma concentrations in the same mice when B16F10 or 4T1 tumour-bearing mice were fed the NSD or HSD for 16 days. The two-tailed Wilcoxon rank-sum tests; n = 10 mice per group; ns, not significant; *p<0.05, **p<0.01 and ***p<0.001 vs. the NSD group. These experiments were repeated three times. Data are representative of three independent experiments and are presented as the mean ± SEM. (d, e) Water content in various organs when 4T1 and B16F10 tumour-bearing mice were fed with the NSD or HSD for 16 days. These experiments were repeated three times. Data are representative of three independent experiments and are presented as the mean ± SEM. The two-tailed Wilcoxon rank-sum tests; n = 10 mice per group; ns, not significant. (f) Na^{+}, K^{+} and Cl^{-} content in B16F10 tumour tissues was determined by X-ray fluorescence spectrometry. The two-tailed Wilcoxon rank-sum tests; n = 10 mice per group; ns, not significant; *p<0.05 and ***p<0.001 vs. the NSD group. These experiments were repeated three times. Data are representative of three independent experiments and are presented as the mean ± SEM. (g) The osmolality

of tissues from B16F10 tumour-bearing mice was examined by vapor pressure osmometry. These experiments were repeated three times. Data are representative of three independent experiments and are presented as the mean ± SEM. The two-tailed Wilcoxon rank-sum tests; n = 10 mice per group; ns, not significant; ***p<0.001 vs. the NSD group. Source data are provided as a Source Data file.

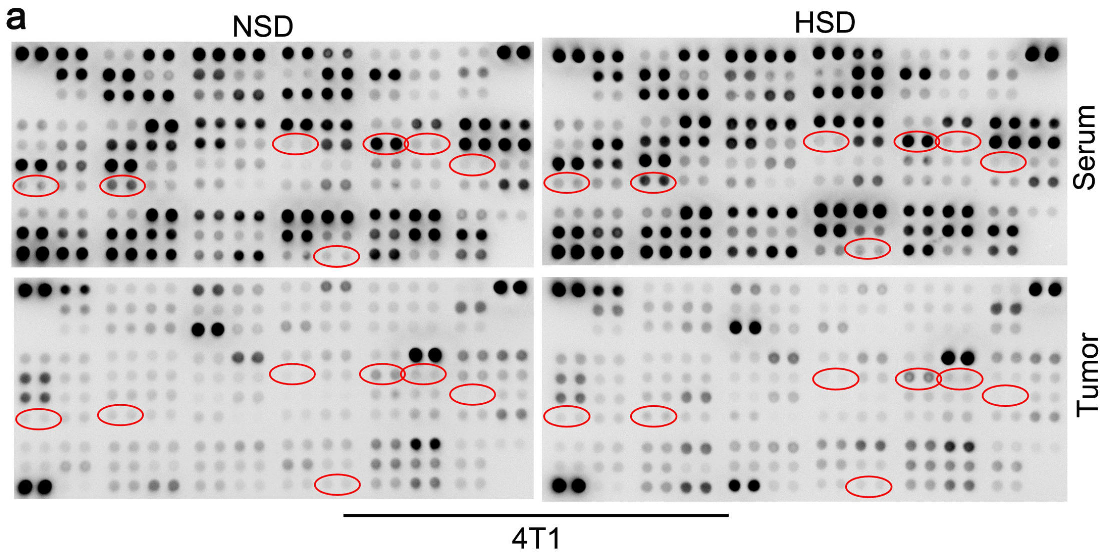

Supplementary Figure 5. The images of inflammatory cytokines detected in tumour tissue lysates and serum. (a) Inflammatory cytokines in tumour tissue lysates and serum from tumour-bearing NSD- or HSD-fed mice were assessed by the Proteome Profiler Mouse XL Cytokine Array, and the relevant upregulated and downregulated cytokines in tumour tissue lysates and serum are indicated by red ovals. n = 5 for NSD group and 6 mice for HSD group. The representative images were from one out of two independent experiments.

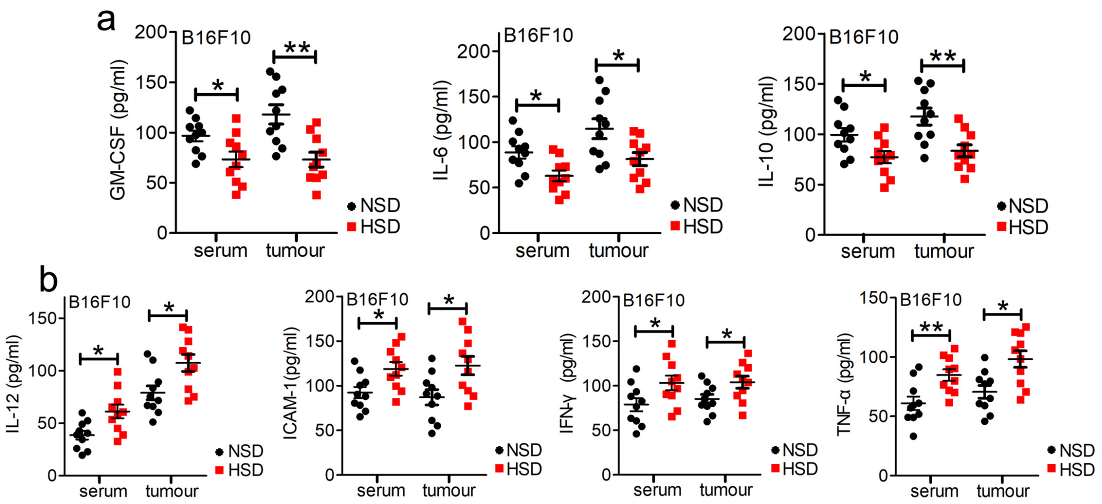

Supplementary Figure 6. The cytokine expression in serum and tumour tissues from B16F10 tumour-bearing mice fed with the NSD or HSD. (a, b) Relative cytokines in serum or tumour tissues from NSD- or HSD-fed B16F10 tumour-bearing mice were analysed by ELISA. For all panels, the two-tailed Wilcoxon rank-sum tests. **p*<0.05 and ***p*<0.01 vs. the NSD group; n = 10 mice per group. Data are from one out of three independent experiments. Data are presented as dot plots extending to minimum and maximum values in one independent experiments and bar are presented as the mean ± SEM of 10 individual mice. Source data are provided as a Source Data file.

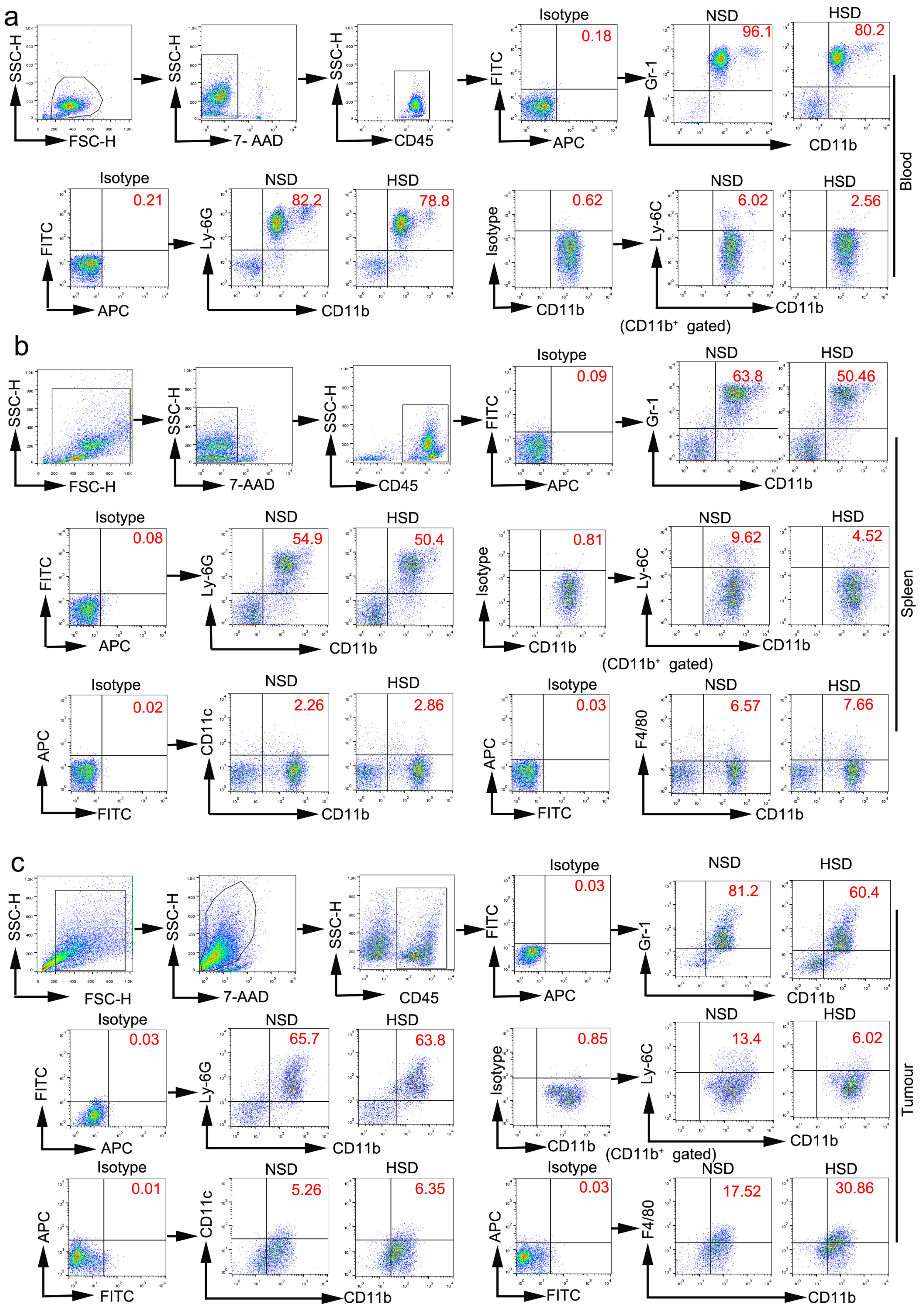

Supplementary Figure 7. Gating strategy for the quantification of MDSCs, macrophages and DCs in the blood, spleen and tumour tissues in 4T1 tumour model. (a) Gating strategy for the quantification of MDSCs in the blood. (b) Gating strategy for the quantification of MDSCs, macrophages and DCs in the spleen. (c) Gating

strategy for the quantification of MDSCs, macrophages and DCs in the tumour tissues. n = 10 mice per group.

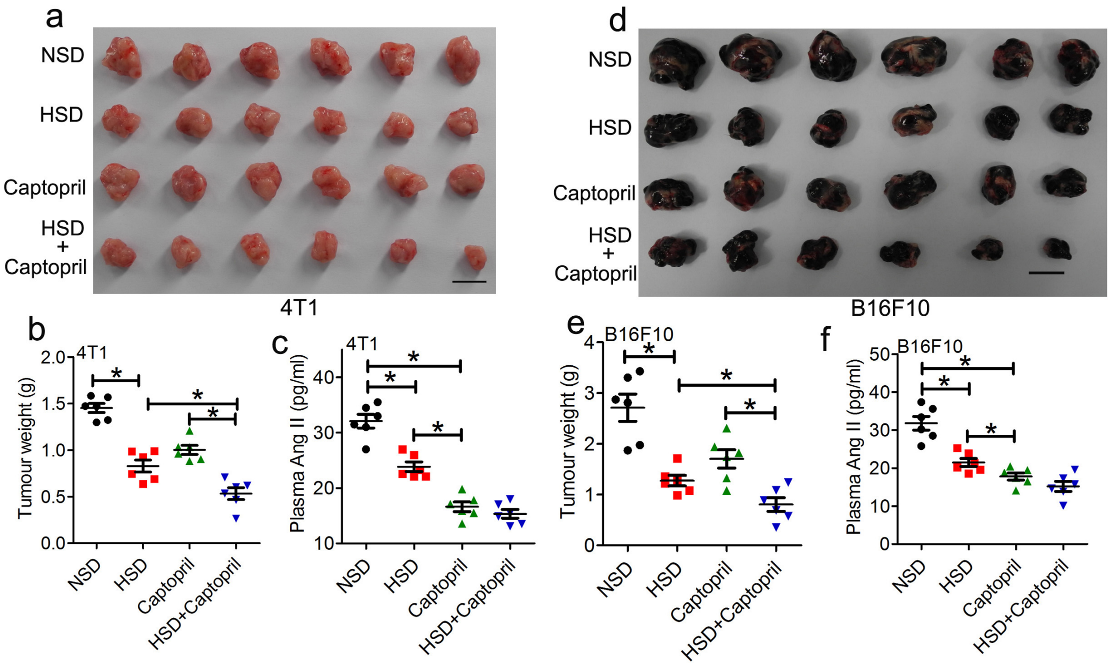

Supplementary Figure 8. The antitumour activity of HSD was independent of RAS blockage *in vivo*. 4T1 and B16F10 tumour-bearing mice were treated with NSD, HSD, captopril or a combination of HSD and captopril. (a, b, d, e) Images of tumours harvested from mice are shown, and tumour size was measured. Scare bar, 1 cm. (c, f) The level of plasma angiotensin II (Ang II) was examined by ELISA. For all panels, the data are expressed as the mean ± SEM; n = 6 mice per group; *$p<0.05$; one-way ANOVA with post hoc Bonferroni correction.Data are presented as dot plots extending to minimum and maximum values in one independent experiments and bar are presented as the mean ± SEM of 6 individual mice; each dot represents one mouse. These experiments were repeated twice. Source data are provided as a Source Data file.

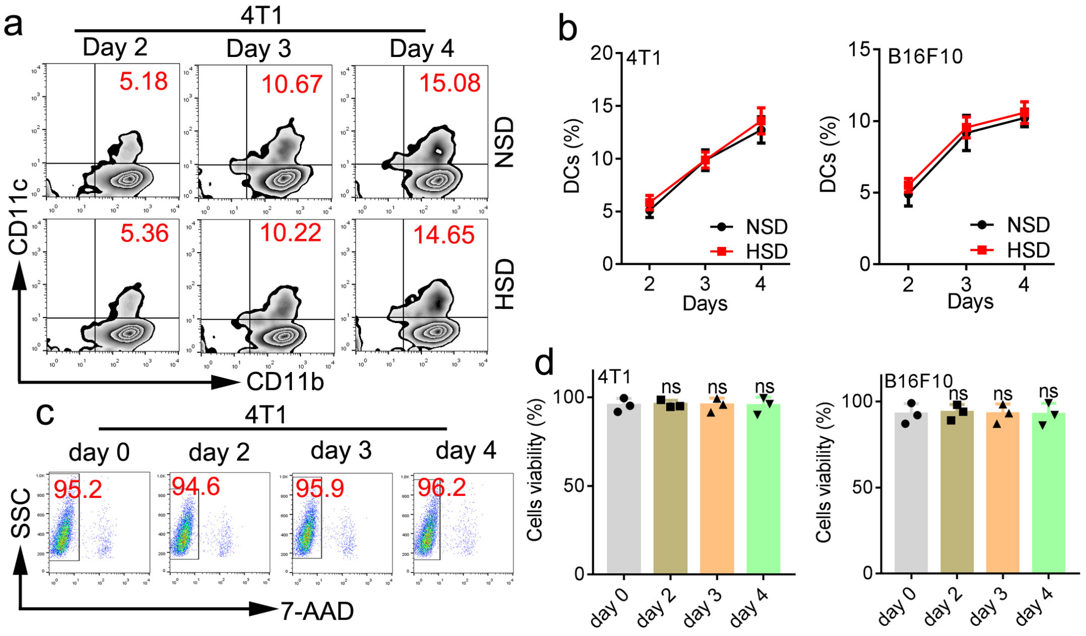

Supplementary Figure 9. HSD did not affect the differentiation of M-MDSCs towards DCs. (a, b) Tumour-infiltrating M-MDSCs from the NSD or HSD group were cultured for 2, 3 and 4 days. The population of CD11b$^+$CD11c$^+$ cells was evaluated by flow cytometry. One-way ANOVA with post hoc Bonferroni correction; n = 10 mice per group; ns, not significant; Bars are expressed as the mean ± SEM of 3 independent replicates with isolated cells pooled from the same group of mice. These experiments were replicated with similar results. (c, d) During M-MDSC differentiation, cell viability was examined using flow cytometry via staining with 7-AAD. One-way ANOVA with Dunnett's test; ns, not significant compared viability at day 0; n = 10 mice per group. Bars are expressed as the mean ± SEM of 3 independent replicates with isolated cells pooled from the same group of mice. These experiments were replicated with similar results. Source data are provided as a Source Data file.

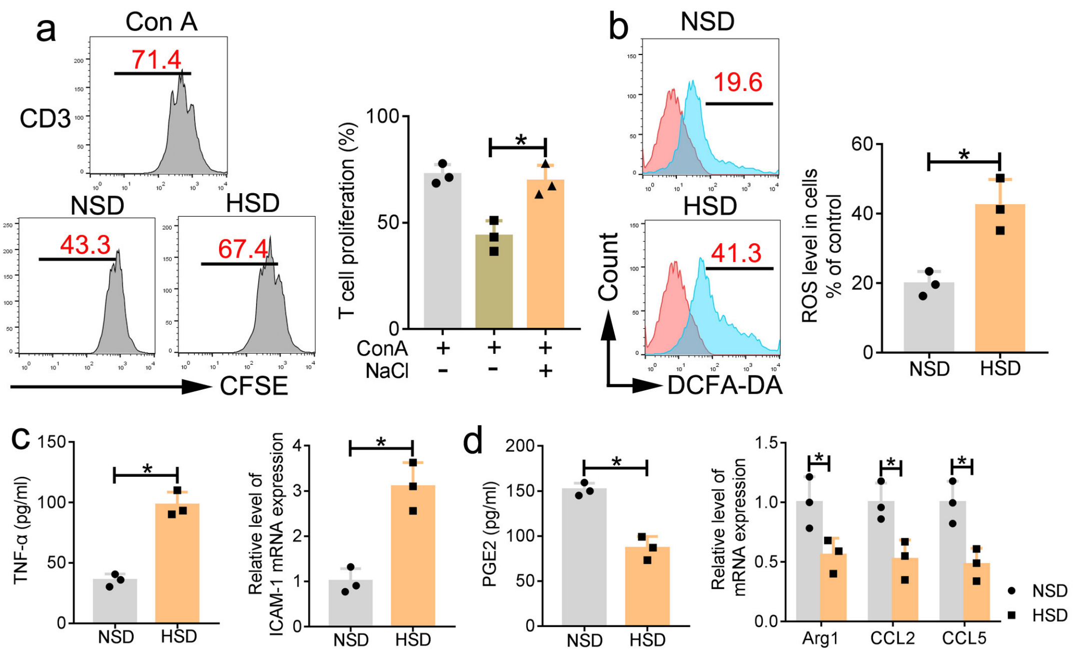

Supplementary Figure 10. HSD promoted a switch in PMN-MDSC function *in vivo*. (a) Effect of HSD on the immunosuppressive activity of tumour-infiltrating PMN-MDSCs. $CD3^+$ T cell proliferation was examined with the CFSE dilution assay after labelled splenocytes were co-cultured with PMN-MDSCs isolated from NSD- or HSD-fed mice (2:1) for 3 days with Con A stimulation. (b) Analysis of ROS production by PMN-MDSCs isolated from tumour tissues from the NSD or HSD group. Cells were incubated with dichlorofluorescein diacetate (DCFA-DA). (c, d) An equal of number of purified tumour-infiltrating PMN-MDSCs from the NSD and HSD groups were cultured in RPMI-1640 medium containing 10% FBS for 24 h. The concentrations of TNF-α and PGE2 in the supernatant were examined by ELISA, and gene expression of ICAM-1, Arg1, CCL2 and CCL5 in PMN-MDSCs was tested by qRT-PCR. For all panels, *p<0.05 vs. the NSD group. Two-tailed Student's *t*-test. Bars are expressed as the mean ± SEM of 3 independent replicates with isolated cells pooled from the same group of mice. These experiments were replicated with similar results; n = 5 for NSD and 6 for HSD mice per group. Source data are provided as a Source Data file.

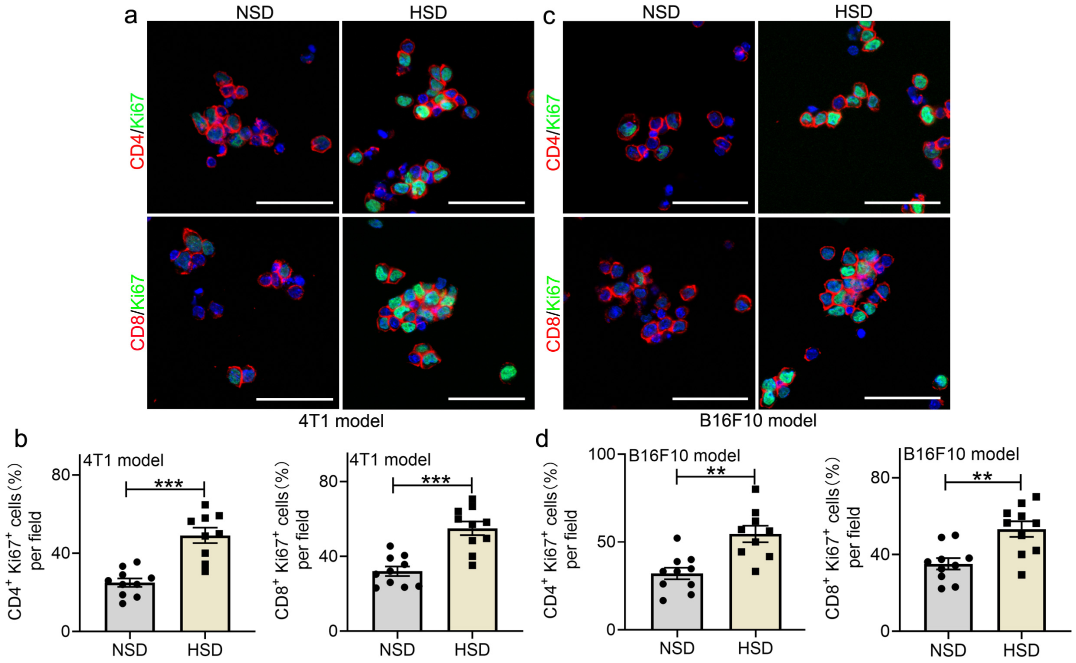

Supplementary Figure 11. The effects of HSD on the expression of Ki67 in T cells isolated from tumour tissues. After isolated from NSD or HSD treated tumour-bearing mice, the expression of Ki67 in $CD4^+$ or $CD8^+$ T cells were evaluated by immunofluorescent imaging (a-d). Green, Ki67; blue, DAPI nuclear staining; red, CD4 or CD8; scale bar, 50 μm. The number of $Ki67^+$ cells in high-power optic (×1000 magnification) field in stained sections (For $CD4^+$ $Ki67^+$ cells per field, n=10 individual tumours in NSD group and n=9 individual tumours in HSD group; For $CD8^+$ $Ki67^+$ cells per field, n=10 individual tumours in NSD and HSD group). The two-tailed Wilcoxon rank-sum tests; n = 10 for NSD and HSD group; $**p<0.01$; $***p<0.001$; Bars are expressed as the mean ± SEM. These experiments were repeated 3 times. Source data are provided as a Source Data file.

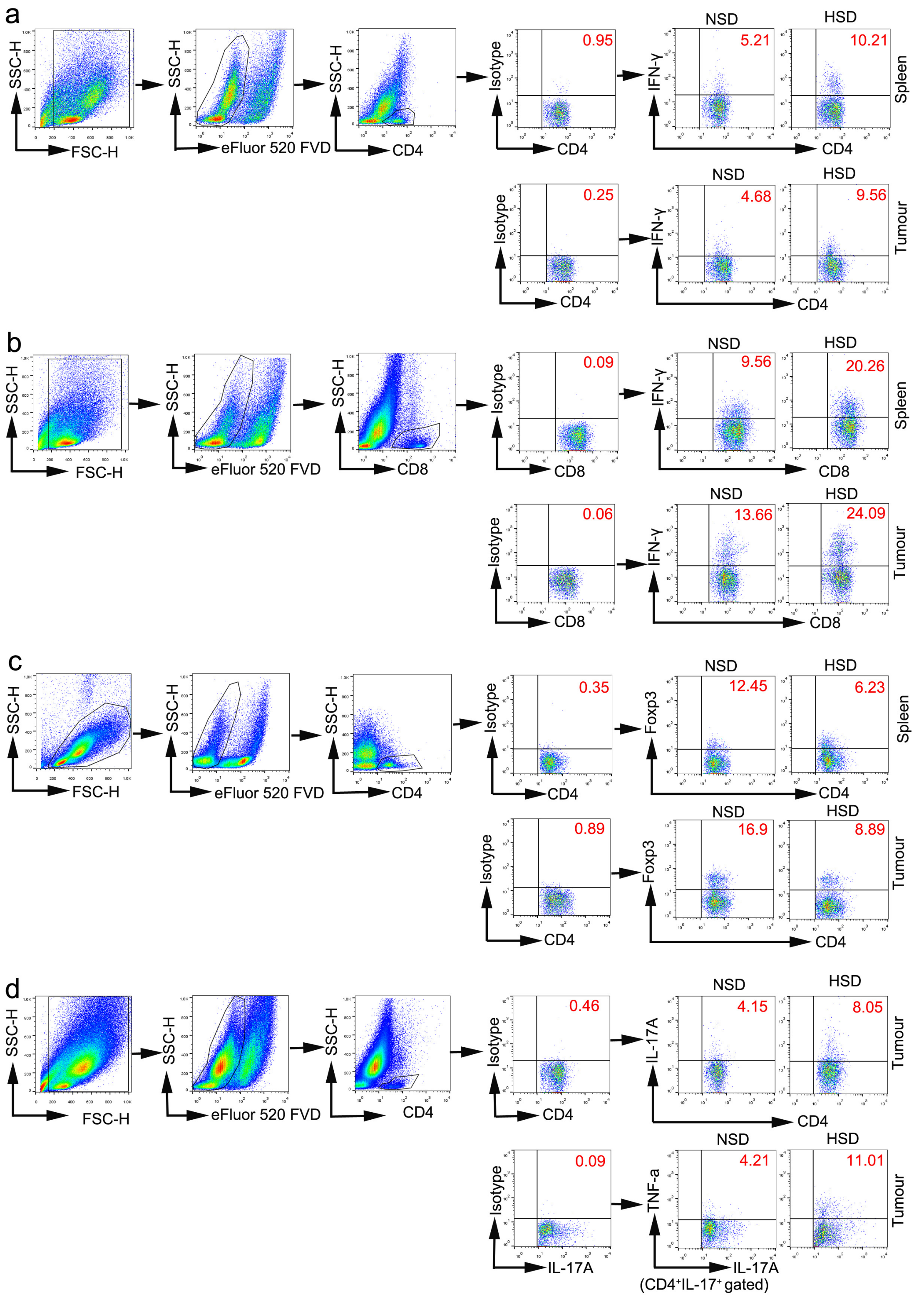

Supplementary Figure 12. Gating strategy for the quantification of IFN-γ$^+$CD4$^+$ cells, IFN-γ$^+$CD8$^+$ cells, CD4$^+$Foxp3$^+$ Treg cells, Th17 cells and TNF-α$^+$ Th17 cells in the spleen and/or tumour tissues in 4T1 tumour model. (a) Gating strategy for the quantification of IFN-γ$^+$CD4$^+$ cells in the spleen and tumour tissues. (b) Gating

strategy for the quantification of IFN-γ$^+$CD8$^+$ cells in the spleen and tumour tissues. (c) Gating strategy for the quantification of CD4$^+$ Foxp3$^+$ Treg cells in the spleen and tumour tissues. (d) Gating strategy for the quantification of Th17 cells and TNF-α$^+$ Th17 cells in the tumour tissues. n = 10 mice per group.

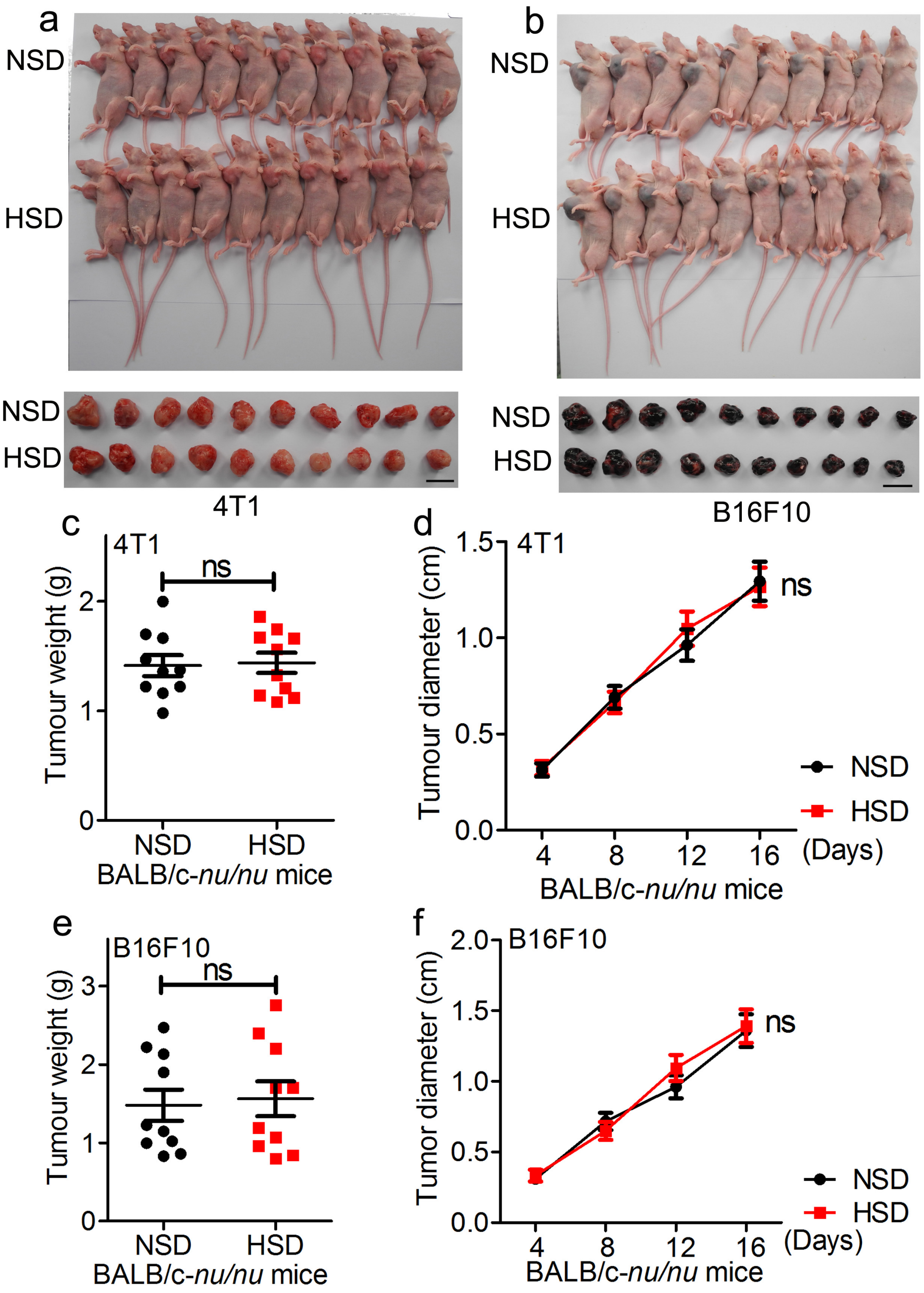

Supplementary Figure 13. HSD displayed antitumour activity through a T cell-mediated immune response. (a-f) Images of tumours harvested from null mice are shown, and tumour weight and size were measured; n = 10 mice per group. The two-tailed Wilcoxon rank-sum tests; ns, not significant. Data are presented as dot plots extending to minimum and maximum values in one independent experiments and bar are presented as the mean ± SEM of 10 individual mice; each dot represents one mouse. These experiments were repeated 2 times. Source data are provided as a Source Data file.

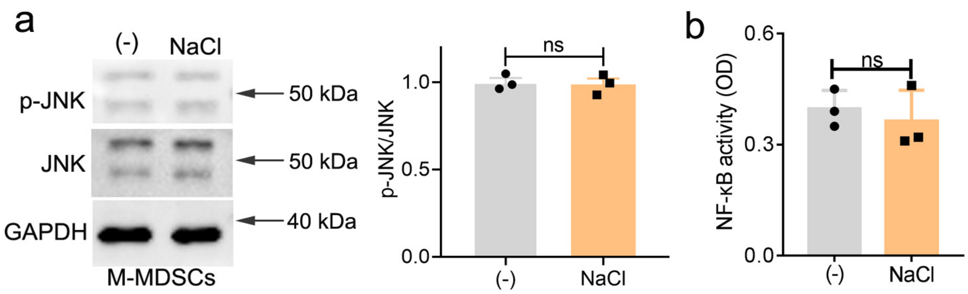

Supplementary Figure 14. The effect of high salt on JNK and NF-κB activity. Isolated M-MDSCs from tumour tissue were stimulated in the presence or absence of an additional 40 mM NaCl for 1 h. p-JNK was analysed by western blotting, and grey level of western blot was analysed by Image J (a); NF-κB activity was determined by the p65 subunit DNA-binding ability (b). These experiments were repeated 3 times; two-tailed Student's *t*-test; n = 6 mice; ns, not significant. The western blotting data are representative of three independent experiments. Bars are expressed as the mean ± SEM of 3 independent replicates with isolated cells pooled from the same group of mice. Source data are provided as a Source Data file.

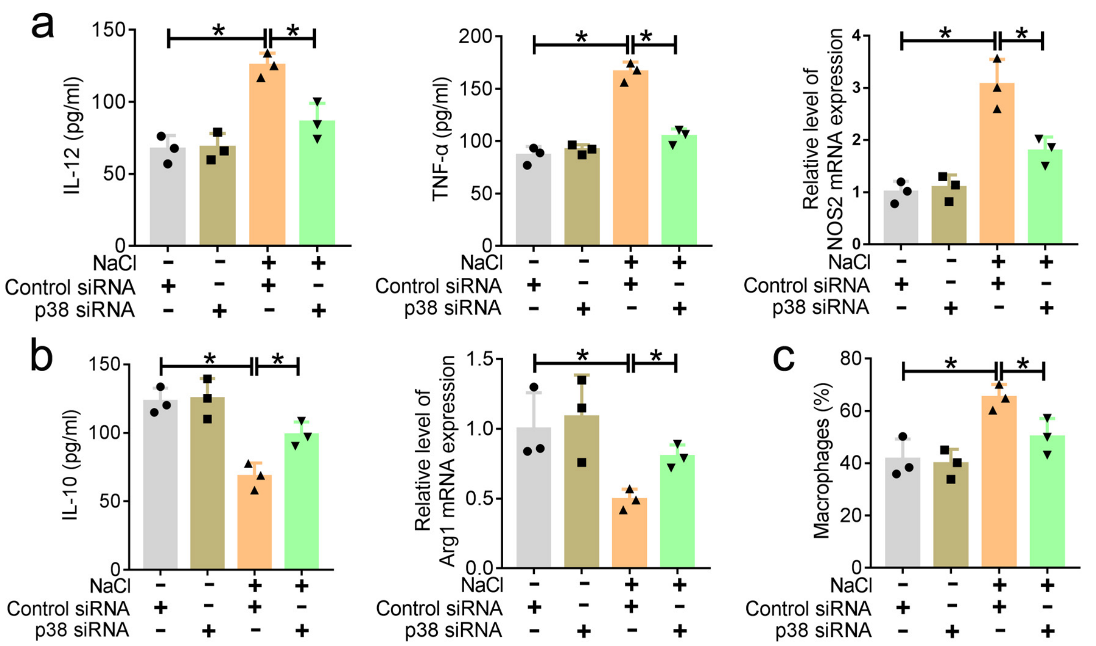

Supplementary Figure 15. p38 silencing reduced HSD-induced M-MDSC differentiation and function *in vitro*. Purified tumour M-MDSCs were transfected with p38-specific siRNA or control siRNA for 2 days and cultured in the absence or presence of an additional 40 mM NaCl for 24 h. (a, b) The expression of IL-12, TNF-α and IL-10 in the supernatant was determined by ELISA, and NOS2 and Arg1 mRNA levels were evaluated by qRT-PCR. (c) The population of $CD11b^{+}F4/80^{+}$ cells were tested by flow cytometry. These experiments were replicated with similar results. n = 5 mice; **p*<0.05, one-way ANOVA with post hoc Bonferroni correction. Bars are expressed as the mean ± SEM of 3 independent replicates with isolated cells pooled from the same group of mice. Source data are provided as a Source Data file.

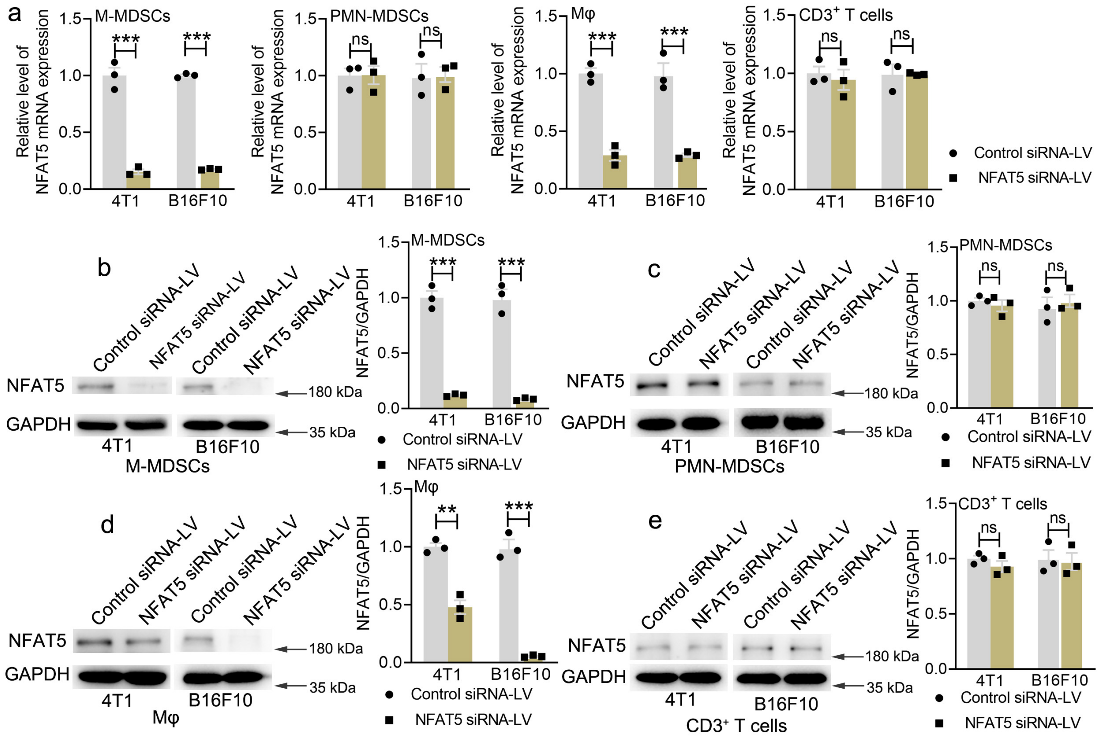

Supplementary Figure 16. The effect of Lentivirus vector injected into tumour-bearing mice on the expression of NFAT5 in immune cells subsets in tumour tissue. 4T1 and B16F10 tumour-bearing mice were injected with lentiviruses siRNA-NFAT5-LV or siRNA-control-LV via i.v. administration every two days. After 16 days, the expression of NFAT5 in purified tumour M-MDSCs, PMN-MDSCS, Macrophage (Mφ) and $CD3^+$ T cells from the above groups was examined by qRT-PCR (a) and western blotting (b-e). Two-tailed Student's t-test; ns, not significant, ***p*<0.01 and ****p*<0.001; n = 6 mice per group. Bars are expressed as the mean ± SEM of 3 independent replicates with isolated cells pooled from the same group of mice. These experiments were replicated with similar results. Source data are provided as a Source Data file.

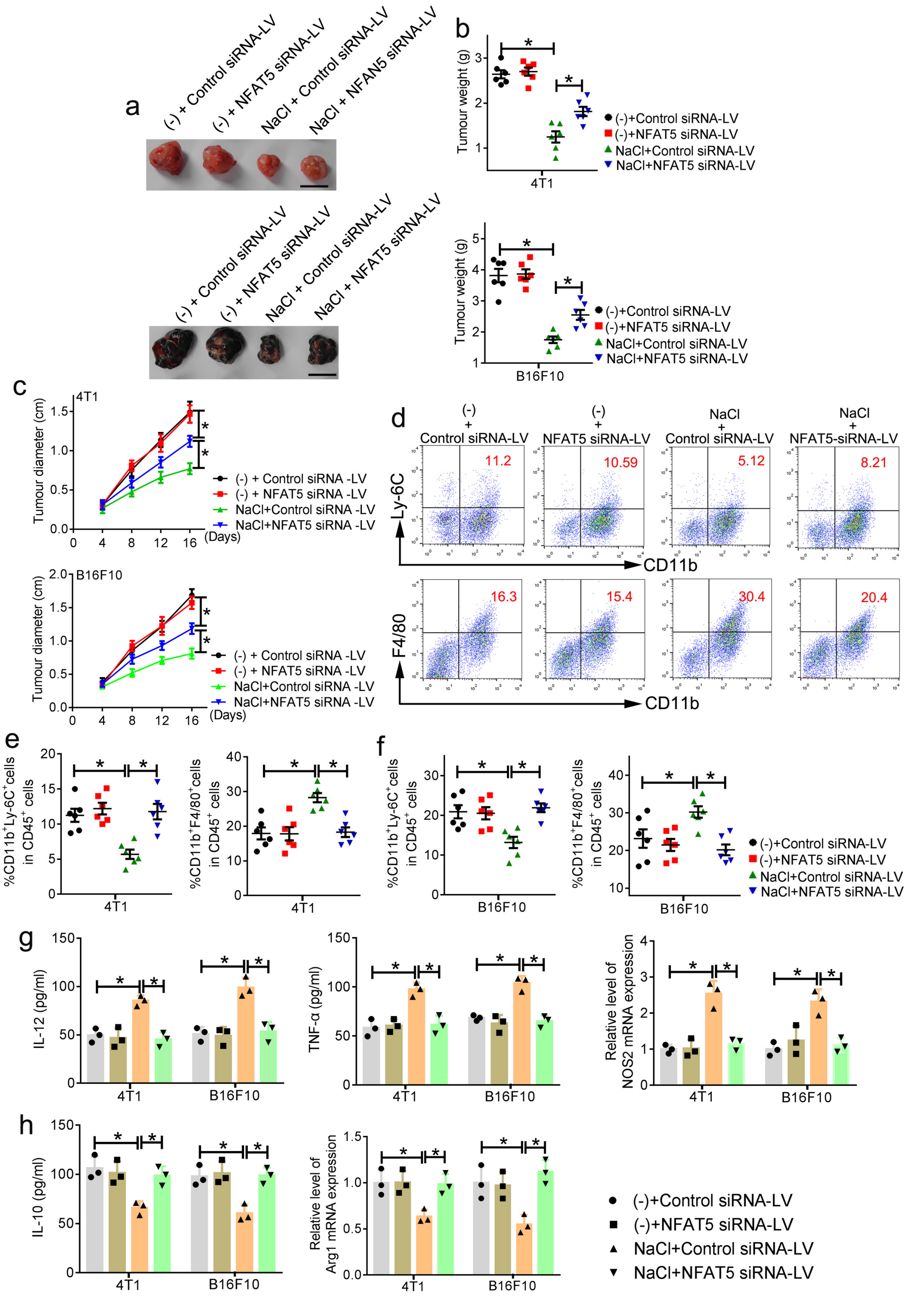

Supplementary Figure 17. NFAT5 deficiency attenuated HSD-induced M-MDSC differentiation and function *in vivo*. (a-c) Images of tumours harvested from 4T1 and B16F10 tumour-bearing mice fed with the NSD or HSD treatment with lentiviruses siRNA-NFAT5-LV or siRNA-control-LV are shown, and tumour weight and size were

measured; n = 6 mice per group. One-way ANOVA with post hoc Bonferroni correction; *$p<0.05$. Data are presented as dot plots extending to minimum and maximum values in one independent experiments and bar are presented as the mean ± SEM of 6 individual mice; each dot represents one mouse. (d-f) Tumours collected at day 16 were dissociated, and the proportions of M-MDSCs ($CD11b^+Ly\text{-}6C^+$) and macrophages ($CD11b^+F4/80^+$) in $CD45^+$ cells were determined by flow cytometry. Data are presented as dot plots extending to minimum and maximum values in one independent experiments and bar are presented as the mean ± SEM of 6 individual mice; each dot represents one mouse. One-way ANOVA with post hoc Bonferroni correction; *$p<0.05$; n = 6 mice per group. (g, h) Purified tumour M-MDSCs from the above groups were cultured 24 h, and the expression of IL-12, TNF-α and IL-10 in the supernatant was determined by ELISA. NOS2 and Arg1 mRNA levels were evaluated by qRT-PCR. Bars are expressed as the mean ± SEM of 3 independent replicates with isolated cells pooled from the same group of mice. These experiments were replicated with similar results. One-way ANOVA with post hoc Bonferroni correction; *$p<0.05$; n = 6 mice per group. Source data are provided as a Source Data file.

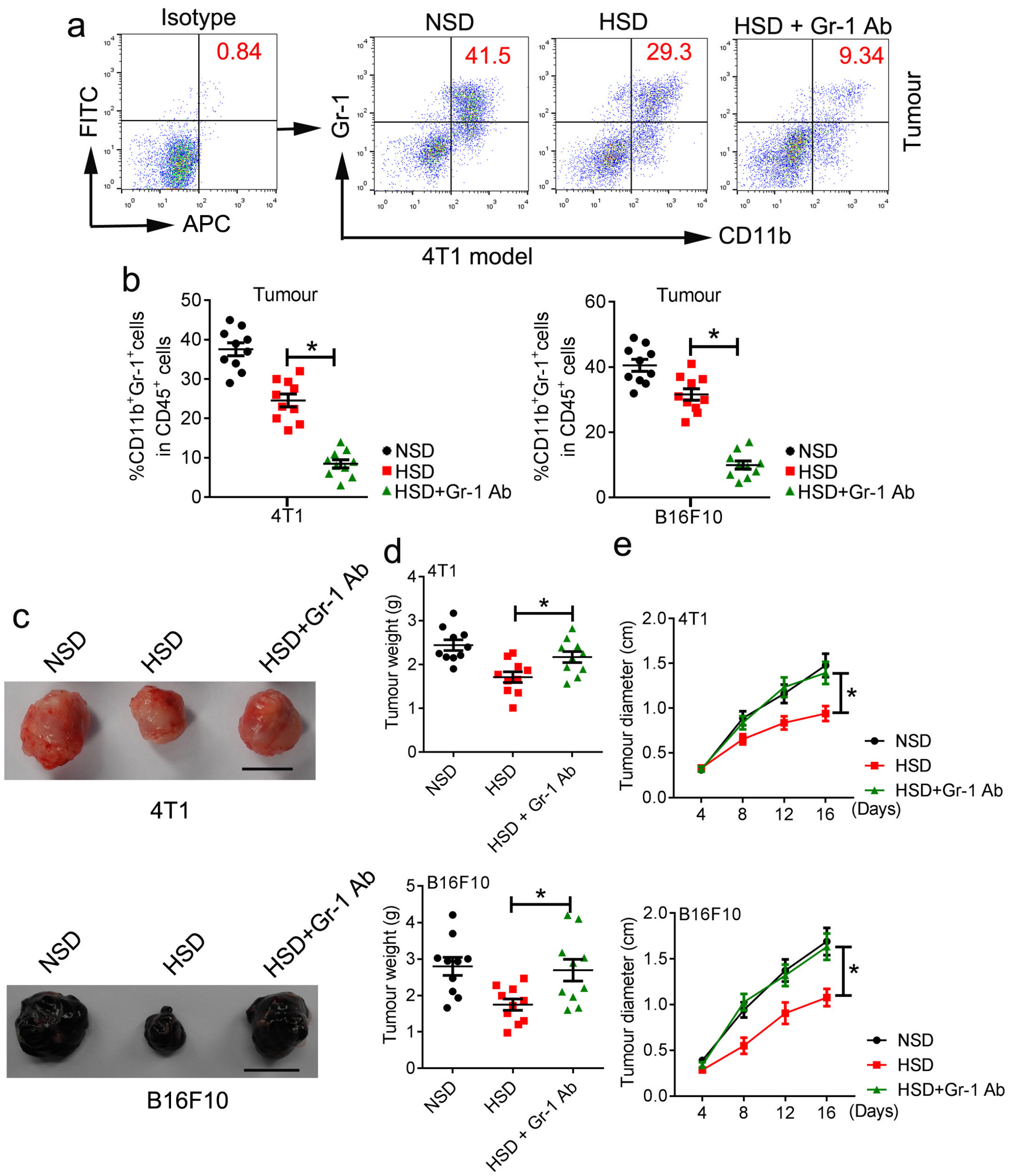

Supplementary Figure 18. MDSC depletion eliminated the anti-tumour activity of HSD. Female mice were subcutaneously implanted with $1×10^6$ 4T1 or B16F10 cells after starvation for 1 day and randomly assigned to receive the NSD or HSD for 16 days. Mice were injected with 200 µg isotype controls or 200 µg anti-Gr-1 monoclonal antibody via intraperitoneal (i.p.) administration every two days (n = 10 for each group). (a, b) Tumours collected at day 16 were dissociated, and the proportions of MDSCs ($CD11b^+$ $Gr-1^+$ cells) in the $CD45^+$ cell population were determined by flow cytometry. (c-e) Images of tumours harvested from mice are shown, and tumour size was examined. For all panels, n = 10 mice per group.

One-way ANOVA with post hoc Bonferroni correction; *p<0.05 vs. the control. Data are presented as dot plots extending to minimum and maximum values in one independent experiments and bar are presented as the mean ± SEM of 10 individual mice; each dot represents one mouse. These experiments were repeated 3 times. Source data are provided as a Source Data file.

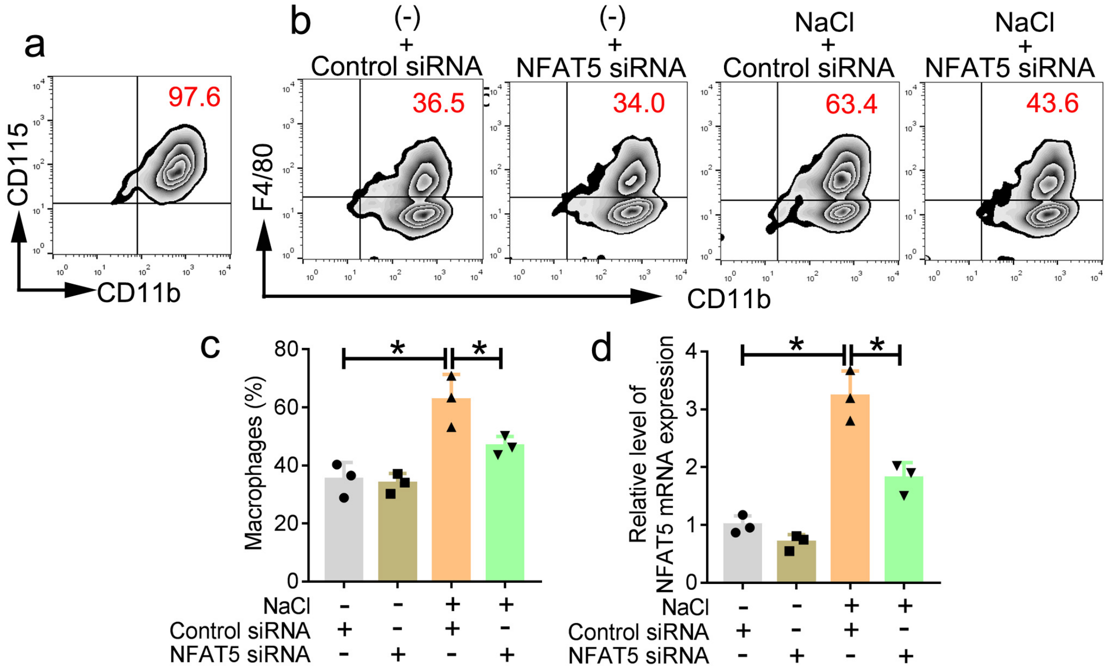

Supplementary Figure 19. HSD promoted the differentiation of normal monocytes into macrophages via NFAT5 expression *in vitro*. (a) The purity of monocytes isolated from blood were examined by double staining with CD11b and CD115 using flow cytometry. After monocytes were transfected with NFAT5 siRNA, cells were stimulated with 10 ng ml^{-1} GM-CSF in the presence of 40 mM NaCl for 3 days. The macrophage proportion was determined by flow cytometry (b, c). The mRNA level of NFAT5 was analysed by qRT-PCR on day 3 (d). One-way ANOVA with post hoc Bonferroni correction; n = 6 mice; *p<0.05. Bars are expressed as the mean ± SEM of 3 independent replicates with isolated cells pooled from the same group of mice. These experiments were repeated 3 times. Source data are provided as a Source Data file.

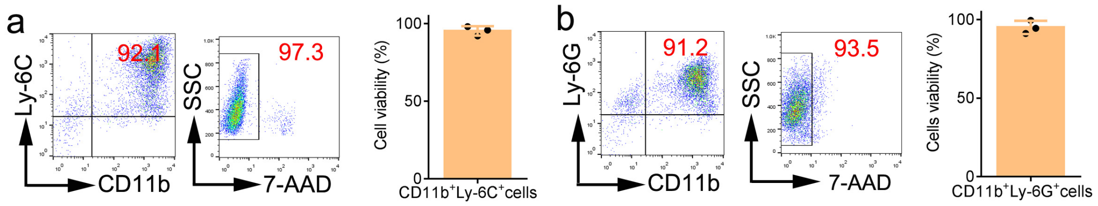

Supplementary Figure 20. Analysis of the purity and viability of isolated M-MDSCs and PMN-MDSCs. (a) After M-MDSCs were isolated from tumour tissues, cells were stained with CD11b and Ly-6C antibodies or 7-AAD, and the proportion of $CD11b^{+}Ly\text{-}6C^{+}$ cells and cell viability were examined by flow cytometry. (b) After PMN-MDSCs were isolated from tumour tissues, cells were stained with CD11b and Ly-6G antibodies or 7-AAD, and the proportion of $CD11b^{+}Ly\text{-}6G^{+}$ cells and cell viability were examined by flow cytometry. n = 6 mice. Bars are expressed as the mean ± SEM of 3 independent replicates with isolated cells pooled from the same group of mice. These experiments were repeated 3 times. Source data are provided as a Source Data file.

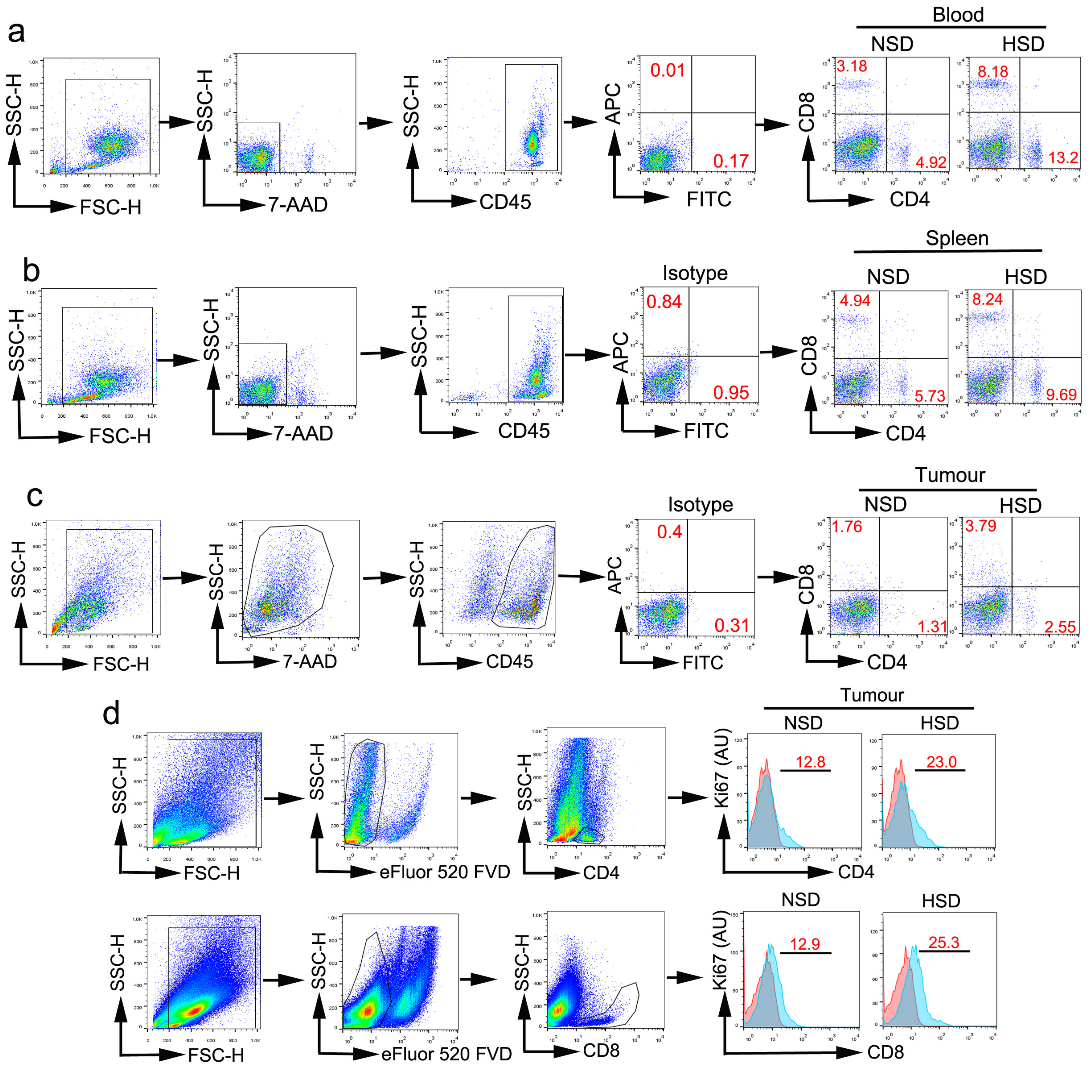

Supplementary Figure 21. Gating strategy for the quantification of CD4⁺ T cells and CD8⁺ T cells in the blood, spleen and tumour tissues as well as analysis of Ki67 expression in CD4⁺, CD8⁺ T cells in tumour tissues. (a) Gating strategy for the quantification of CD4⁺ T cells and CD8⁺ T cells in the blood. (b) Gating strategy for the quantification of CD4⁺ T cells and CD8⁺ T cells in the spleen. (c) Gating strategy for the quantification of CD4⁺ T cells and CD8⁺ T cells in the tumour tissues. (d) Gating strategy for analysis of Ki67 expression in CD4⁺, CD8⁺ T cells in tumour tissues. n = 10 mice per group.

Supplementary Table 1. The antibodies used for western blotting and immunofluorescence analysis are shown.

Antibody	Manufacturer	Dilution
Anti-mouse NFAT5	Abcam (ab3446)	1:1000
Anti-mouse p-p38	Cell Signaling Technology Inc. (#4511)	1:1000
Anti-mouse p38	Cell Signaling Technology Inc. (#8690)	1:1000
Anti-mouse p-JNK	Cell Signaling Technology Inc. (#4668)	1:1000
Anti-mouse JNK	Cell Signaling Technology Inc. (#9252)	1:1000
Anti-mouse Ki67	Cell Signaling Technology Inc. (#9129)	1:200
Anti-mouse CD31	Santa Cruz Biotechnology (sc-1506)	1:100
Anti-mouse F4/80	Abcam (ab6640)	1:100
Anti-mouse IL-10	Boster (BA4317-2)	1:100
Anti-mouse IL-12	Bioss (bs-0767R)	1:100
Anti-mouse CD4	Biolegend (Cat# 100401)	1:100
Anti-mouse CD8	Biolegend (Cat# 100801)	1:100
HRP-conjugated anti-GAPDH	KangChen Bio-tech Inc. (KG-5G5)	1:10000
HRP-conjugated goat anti-rabbit IgG	Jackson ImmunoResearch (111-005-003)	1:2000
Alexa Fluor 546-donkey anti-Rabbit IgG (H+L)	Life (#A10040)	1:200
Alexa Fluor 488-goat anti-Rat IgG (H+L)	Life (#A-11006)	1:200

Supplementary Table 2. The primer sequences used for qRT-PCR are shown.

Name of primer	Sequence (5’ to 3’)
NFAT5-5’	CAGCCAAAAGGGAACTGGAG
NFAT5-3’	GAAAGCCTTGCTGTGTTCTG
ATF2-5’	TCCTCCGGGGCTAGTTTGTA
ATF2-3’	GCAATACTTCCCAAGTTGCCA
Arg1-5’	CTCCAAGCCAAAGTCCTTAGAG
Arg1-3’	AGGAGCTGTCATTAGGGACATC
NOS2-5’	CCAAGCCCTCACCTACTTCC
NOS2-3’	CTCTGAGGGCTGACACAAGG
ICAM-1-5’	GAAGCTTCTTTTGCTCTGCC
ICAM-1-3’	AGCAGTACTGGCACCAGAAT
CCL2-5’	AGCCAACTCTCACTGAAGCC
CCL2-3’	GGACCCATTCCTTCTTGGGG
CCL5-5’	TGCTCCAATCTTGCAGTCGT
CCL5-3’	GCAAGCAATGACAGGGAAGC
TNF-a-5’	TCTCAGGCCTTCCTACCTTCA
TNF-a-3’	GCTCAGCTCCGTTTTCACAGA
IFN-γ-5’	ATTGCGGGGTTGTATCTGGG
IFN-γ-3’	GGAAGCACCAGGTGTCAAGT
β-actin-5’	GGTGTGATGGTGGGAATGGG
β-actin-3’	ACGGTTGGCCTTAGGGTTCAG

Supplementary Table 3. The main ingredients and caloric content in NSD and HSD.

Ingredients (in g kg^{-1} chow)		
	NSD	HSD
Wheat	350.0	350.0
Corn	309.69	263.99
Soybean Meal (48%)	190.8	197.0
Corn Gluten Meal (60%)	50.0	52.0
Alfalfa Meal (17%), dehydrated	30.0	30.0
Corn Oil	33.0	34.5
Sodium Chloride	4.0	40.0
Dicalcium Phosphate, FG (18.5% P, 21% Ca)	14.0	14.0
Calcium Carbonate, FG (38%)	12.0	12.0
Mineral Mix, TSD (80318)	1.5	1.5
Vitamin Mix, TSD (81125)	3.0	3.0
DL-Methionine, FG (99%)	1.0	1.0
L-Lysine HCl, FG (78%)	1.0	1.0
Ethoxyquin, antioxidant	0.01	0.01
Caloric content (kcal g^{-1} chow)	3.2	3.1

Supplementary Table 4. The antibodies used for flow cytometry are shown.

Antibody	Manufacturer	Dilution
APC anti-mouse CD11b	Biolegend (Cat# 101212)	1:100
FITC anti-mouse Gr-1	Biolegend (Cat# 108406)	1:200
PE anti-mouse CD45	Biolegend (Cat# 103105)	1:400
FITC anti-mouse Ly-6C	Biolegend (Cat# 128006)	1:200
FITC anti-mouse Ly-6G	Biolegend (Cat# 127605)	1:200
FITC anti-mouse CD4	Biolegend (Cat# 100510)	1:200
APC anti-mouse CD8	Biolegend (Cat# 100712)	1:100
APC anti-mouse F4/80	Biolegend (Cat# 123115)	1:100
FITC anti-mouse CD11b	Biolegend (Cat# 101205)	1:200
APC anti-mouse CD11c	Biolegend (Cat# 117310)	1:100
APC anti-mouse Ki67	Biolegend (Cat# 652405)	1:100
PE anti-mouse CD8a	Biolegend (Cat# 100708)	1:400
APC anti-mouse IFN-γ	Biolegend (Cat# 505809)	1:100
Alexa Fluor 647 anti-mouse TNF-α	Biolegend (Cat# 506314)	1:100
PE anti-mouse IL-17A	Biolegend (Cat# 506903)	1:400
PE/Cy7 anti-mouse CD4	Biolegend (Cat# 100421)	1:100
PE/Cy7 anti-mouse CD8a	Biolegend (Cat# 100721)	1:100
PE anti-mouse Foxp3	Biolegend (Cat# 126403)	1:100
APC anti-mouse CD4	Biolegend (Cat# 100411)	1:100
PE anti-mouse Gr-1	Biolegend (Cat# 108408)	1:400
APC rat IgG2b, κ isotype control antibody	Biolegend (Cat# 400611)	1:100
FITC rat IgG2b, κ isotype control antibody	Biolegend (Cat# 400605)	1:200
PE rat IgG2b, κ isotype control antibody	Biolegend (Cat# 400608)	1:400
FITC rat IgG2c, κ isotype control antibody	Biolegend (Cat# 400705)	1:200
FITC rat IgG2a, κ isotype control antibody	Biolegend (Cat# 400505)	1:200
APC rat IgG2a, κ isotype control antibody	Biolegend (Cat# 400512)	1:100
FITC rat IgG2b, κ isotype control antibody	Biolegend (Cat# 400633)	1:200
APC Armenian hamster IgG isotype control	Biolegend (Cat# 400912)	1:100

Antibody		
APC rat IgG1, κ isotype control antibody	Biolegend (Cat# 400412)	1:100
PE rat IgG2a, κ isotype control antibody	Biolegend (Cat# 400507)	1:400
PE rat IgG1, κ isotype control antibody	Biolegend (Cat# 400407)	1:400
PE/Cy7 Rat IgG2a, κ Isotype control antibody	Biolegend (Cat# 400521)	1:100
PE/Cy7 Rat IgG2b, κ Isotype control antibody	Biolegend (Cat# 400617)	1:100
Alexa Fluor 647 Rat IgG1, κ isotype control antibody	Biolegend (Cat# 400418)	1:100
7-AAD Viability Staining Solution	Biolegend (Cat# 420404)	1:100
Fixable Viability Dye eFluor™ 520	eBioscience(Cat#65-0867-18)	1:1000

Supplementary Table 5. A complete list of multiple cytokines, chemokines, growth factors and other soluble proteins in Proteome Profiler Mouse XL Cytokine Array

	1 2	3 4	5 6	7 8	9 10	11 12	13 14	15 16	17 18	19 20	21 22	23 24
A	Refrence Spots	Adiponectin/Acrp30	Amphiregulin	Angiopoietin-1	Angiopoietin-2	Angiopoietin-like 3	BAFF/BLyS/TNFSF13B	C1q R1/CD93	CCL2/JE/MCP-1	CCL3/CCL4/MIP-1α/β	CCL5/RANTES	Reference Spots
B		CCL6/C10	CCL11/Eotaxin	CCL12/MCP-5	CCL17/TARC	CCL19/MIP-3β	CCL20/MIP-3α	CCL21/6Ckine	CCL22/MDC	CD14	CD40/TNFRSF5	
C		CD160	Chemerin	Chitinase 3-like 1	Coagulation Factor III/Tissue Factor	Complement Component C5/C5a	Complement Factor D	C-Reactive Protein/CRP	CX3CL1/Fractalkine	CXCL1/KC	CXCL2/MIP-2	
D	CXCL9/MIG	CXCL10/IP-10	CXCL11/I-TAC	CXCL13/BLC/BCA-1	CXCL16	Cystatin C	DKK-1	DPPIV/CD26	EGF	Endoglin/CD105	Endostatin	Fetuin A/AHSG
E	FGF acidic	FGF-21	Flt-3 Ligand	Gas 6	G-CSF	GDF-15	GM-CSF	HGF	ICAM-1/CD54	IFN-γ	IGFBP-1	IGFBP-2
F	IGFBP-3	IGFBP-5	IGFBP-6	IL-1α/IL-1F1	IL-1β/IL-1F2	IL-1ra/IL-1F3	IL-2	IL-3	IL-4	IL-5	IL-6	IL-7
G	IL-10	IL-11	IL-12 p40	IL-13	IL-15	IL-17A	IL-22	IL-23	IL-27 p28	IL-28A/B	IL-33	LDL R
H	Leptin	LIF	Lipocalin-2/NGAL	LIX	M-CSF	MMP-2	MMP-3	MMP-9	Myeloperoxidase	Osteopontin (OPN)	Osteoprotegerin/TNFRSF11B	PD-ECGF/Thymidine phosphorylase
I	PDGF-BB	Pentraxin 2/SAP	Pentraxin 3/TSG-14	Periostin/OSF-2	Pref-1/DLK-1/FA1	Proliferin	Proprotein Convertase 9/PCSK9	RAGE	RBP4	Reg3G	Resistin	
J	Reference Spots	E-Selectin/CD62E	P-Selectin/CD62P	Serpin E1/PAI-1	Serpin F1/PEDF	Thrombopoietin	TIM-1/KIM-1/HAVCR	TNF-α	VCAM-1/CD106	VEGF	WISP-1/CCN4	Negative Control
