# Supplementary material for: High-salt diet inhibits tumour growth in mice via regulating myeloid-derived suppressor cell differentiation
Source: Nat Commun. 2020 Apr 7;11:1732. doi: 10.1038/s41467-020-15524-1 (PMC7138858; doi:10.1038/s41467-020-15524-1)
Supplement: Supplementary file 3 — Reporting Summary [file 41467_2020_15524_MOESM3_ESM.pdf]

nature research

Corresponding author(s): Lei Dong

Last updated by author(s): Feb 10, 2020

Reporting Summary

Nature Research wishes to improve the reproducibility of the work that we publish. This form provides structure for consistency and transparency in reporting. For further information on Nature Research policies, see Authors & Referees and the Editorial Policy Checklist.

Statistics

For all statistical analyses, confirm that the following items are present in the figure legend, table legend, main text, or Methods section.

n/a	Confirmed	
☐	☒	The exact sample size (*n*) for each experimental group/condition, given as a discrete number and unit of measurement
☐	☒	A statement on whether measurements were taken from distinct samples or whether the same sample was measured repeatedly
☐	☒	The statistical test(s) used AND whether they are one- or two-sided<br>*Only common tests should be described solely by name; describe more complex techniques in the Methods section.*
☒	☐	A description of all covariates tested
☐	☒	A description of any assumptions or corrections, such as tests of normality and adjustment for multiple comparisons
☐	☒	A full description of the statistical parameters including central tendency (e.g. means) or other basic estimates (e.g. regression coefficient) AND variation (e.g. standard deviation) or associated estimates of uncertainty (e.g. confidence intervals)
☐	☒	For null hypothesis testing, the test statistic (e.g. *F*, *t*, *r*) with confidence intervals, effect sizes, degrees of freedom and *P* value noted<br>*Give P values as exact values whenever suitable.*
☒	☐	For Bayesian analysis, information on the choice of priors and Markov chain Monte Carlo settings
☒	☐	For hierarchical and complex designs, identification of the appropriate level for tests and full reporting of outcomes
☐	☒	Estimates of effect sizes (e.g. Cohen's *d*, Pearson's *r*), indicating how they were calculated

Our web collection on statistics for biologists contains articles on many of the points above.

Software and code

Policy information about availability of computer code

Data collection

1. WB data were collected by Tanon-4200SF.
2. QPCR data were collected by ABI PRISM 7300 Sequence Detection System.
3.Haematoxylin and eosin (H&E) staining and immunofluorescence staining were captured using Nikon confocal microscope.
4. Cytokine antibody array data were collected by luminescence detector (Chemi Scope 6300).
5. Na+ and K+ content and Cl- content were collected by atomic adsorption spectrometry (HITACHI180-80) or titration with 0.1N silver nitrate (Model Titrando, German Metrohm).
6. The Na+, K+ and Cl- content of tumour samples was assayed by X-ray fluorescence spectrometry at the Center of Modern Analysis (Nanjing University).
7. Tissue osmolality was examined by using a vapor pressure osmometer (Vapro 5520, Wescor, logan, UT).
8. The whole-blood osmolality measurements were identical to those of serum, which was measured by a STY-1A pressure osmometer (TDTF, Tianjing, China).
9. .Mircoarry data was acquired by Agilent DNA Microarray Scanner (Cat.# G2505C, Agilent technologies, Santa Clara, CA, US)

Data analysis

1.Flow cytometry data were analyzed using FlowJo V10.
2.Mircoarry data were extracted with Agilent Feature Extraction software 11.0.1.1 (Agilent technologies, Santa Clara, CA, US). Raw data were normalized by the GeneSpring GX v12.1 software package, KEGG Pathway analysis were performed in the standard enrichment computation method according to the KEGG database (https://www.genome.jp/kegg).
3.Statistical analysis was performed using Graphpad Prism 5 or 7, after after the all data sets for normal distribution were tested by IBM SPSS Statistics 20 software (IBM Corp., Armonk, NY, USA).

For manuscripts utilizing custom algorithms or software that are central to the research but not yet described in published literature, software must be made available to editors/reviewers. We strongly encourage code deposition in a community repository (e.g. GitHub). See the Nature Research guidelines for submitting code & software for further information.

Data

Policy information about availability of data

All manuscripts must include a data availability statement. This statement should provide the following information, where applicable:

- Accession codes, unique identifiers, or web links for publicly available datasets
- A list of figures that have associated raw data
- A description of any restrictions on data availability

All of the data are accessible in the source data file. Data have been deposited in the Gene Expression Omnibus (GEO)/NCBI public database (accession no. GSE125430); The WB raw data is attached in source data.

Field-specific reporting

Please select the one below that is the best fit for your research. If you are not sure, read the appropriate sections before making your selection.

☒ Life sciences ☐ Behavioural & social sciences ☐ Ecological, evolutionary & environmental sciences

For a reference copy of the document with all sections, see nature.com/documents/nr-reporting-summary-flat.pdf

Life sciences study design

All studies must disclose on these points even when the disclosure is negative.

Sample size	Sample size was determined empirically for sufficient statistical power. Variations between samples were also used to determine the suitability of the sample size.
Data exclusions	No data were excluded.
Replication	All attempts at replication were successful.
Randomization	In the subcutaneous implantation tumour model, mice were subcutaneously injected with 1×106 cells (B16F10 or 4T1) into the left armpit after 1 day of starvation. Then, mice were randomly assigned to different groups (5 or 6 mice/ group or 10 mice/group) via random lottery:
Blinding	We needed to investigate the difference between different groups, and such difference had not been known. Thus we did not use blinding in the study.

Reporting for specific materials, systems and methods

We require information from authors about some types of materials, experimental systems and methods used in many studies. Here, indicate whether each material, system or method listed is relevant to your study. If you are not sure if a list item applies to your research, read the appropriate section before selecting a response.

Materials & experimental systems

n/a	Involved in the study	
☐	☒	Antibodies
☐	☒	Eukaryotic cell lines
☒	☐	Palaeontology
☐	☒	Animals and other organisms
☒	☐	Human research participants
☒	☐	Clinical data

Methods

n/a	Involved in the study	
☒	☐	ChIP-seq
☐	☒	Flow cytometry
☒	☐	MRI-based neuroimaging

Antibodies

Antibodies used

1. Anti-mouse NFAT5, Abcam (ab3446)
2. Anti-mouse p-p38, Cell Signaling Technology Inc. (#4511)
3. Anti-mouse p38 ,Cell Signaling Technology Inc. (#8690)
4. Anti-mouse p-JNK, Cell Signaling Technology Inc. (#4668)
5. Anti-mouse JNK ,Cell Signaling Technology Inc. (#9252)
6. Anti-mouse CD31 ,Santa Cruz Biotechnology (sc-1506)
7. Anti-mouse F4/80 ,Abcam (ab6640)
8. Anti-mouse IL-10 ,Boster (BA4317-2)
9. Anti-mouse IL-12 ,Bioss (bs-0767R)
10.Anti-mouse Ki67, Cell Signaling Technology Inc. (#9129)
11.Anti-mouse CD4 Biolegend (Cat# 100401)

12.Anti-mouse CD8 Biolegend (Cat# 100801)
13. HRP-conjugated anti-GAPDH ,KangChen Bio-tech Inc.(KG-5G5)
14. HRP-conjugated goat anti-rabbit IgG, Jackson ImmunoResearch (111-005-003)
15. Alexa Fluor 546-donkey anti-Rabbit IgG (H+L), Life (#A10040)
16. Alexa Fluor 488-goat anti-Rat IgG (H+L), Life (#A-11006)
17. APC anti-mouse CD11b, Biolegend (Cat# 101212)
18. FITC anti-mouse Gr-1, Biolegend (Cat# 108406)
19. PE anti-mouse CD45, Biolegend (Cat# 103105)
20. FITC anti-mouse Ly-6C, Biolegend (Cat# 128006)
21. FITC anti-mouse Ly-6G, Biolegend (Cat# 127605)
22. FITC anti-mouse CD4, Biolegend (Cat# 100510)
23. APC anti-mouse CD8, Biolegend (Cat# 100712)
24. APC anti-mouse F4/80, Biolegend (Cat# 123115)
25. FITC anti-mouse CD11b, Biolegend (Cat# 101205)
26. APC anti-mouse CD11c, Biolegend (Cat# 117310)
27. APC anti-mouse Ki67, Biolegend (Cat# 652405)
28. PE anti-mouse CD8a, Biolegend (Cat# 100708)
29. APC anti-mouse IFN-γ, Biolegend (Cat# 505809)
30. Alexa Fluor 647 anti-mouse TNF-α, Biolegend (Cat# 506314)
31. PE anti-mouse IL-17A, Biolegend (Cat# 506903)
32. PE/Cy7 anti-mouse CD4, Biolegend (Cat# 100421)
33. PE/Cy7 anti-mouse CD8a, Biolegend (Cat# 100721)
34. PE anti-mouse Foxp3, Biolegend (Cat# 126403)
35. APC anti-mouse CD4, Biolegend (Cat# 100411)
36. PE anti-mouse Gr-1, Biolegend (Cat# 108408)
37. APC rat IgG2b, κ isotype control antibody, Biolegend (Cat# 400611)
38. FITC rat IgG2b, κ isotype control antibody, Biolegend (Cat# 400605)
39. PE rat IgG2b, κ isotype control antibody, Biolegend (Cat# 400608)
40. FITC rat IgG2c, κ isotype control antibody, Biolegend (Cat# 400705)
41. FITC rat IgG2a, κ isotype control antibody, Biolegend (Cat# 400505)
42. APC rat IgG2a, κ isotype control antibody, Biolegend (Cat# 400512)
43. FITC rat IgG2b, κ isotype control antibody, Biolegend (Cat# 400633)
44. APC Armenian hamster IgG isotype control Antibody, Biolegend (Cat# 400912)
45. APC rat IgG1, κ isotype control antibody, Biolegend (Cat# 400412)
46. PE rat IgG2a, κ isotype control antibody, Biolegend (Cat# 400507)
47. PE rat IgG1, κ isotype control antibody, Biolegend (Cat# 400407)
48. PE/Cy7 Rat IgG2a, κ Isotype control antibody, Biolegend (Cat# 400521)
49. PE/Cy7 Rat IgG2b, κ Isotype control antibody, Biolegend (Cat# 400617)
50. Alexa Fluor 647 Rat IgG1, κ isotype control antibody, Biolegend (Cat# 400418)

Validation

Antibodies 1-16 was used for the WB or IF staining, and Antibodies 17-50 was used for for flow cytometry. All the antibodies are widely published and have quality control (QC) tested per the company's standard procedure.

Eukaryotic cell lines

Policy information about cell lines

Cell line source(s)

B16F10 (TCM36) and 4T1 (TCM32) cell lines were obtained from the Shanghai Cell Bank of Chinese Academy of Sciences (Shanghai, China).

Authentication

B16F10 and 4T1 cell lines were obtained from the Shanghai Cell Bank of Chinese Academy of Sciences, which have been authenticated.

Mycoplasma contamination

All of the cell lines are negative for mycoplasma contamination.

Commonly misidentified lines
(See ICLAC register)

No commonly misidentified cell lines were used.

Animals and other organisms

Policy information about studies involving animals; ARRIVE guidelines recommended for reporting animal research

Laboratory animals

Female and male C57BL/6J and BALB/C mice (6-8 weeks old) and female BABL/C-nu/nu mice (6-8 weeks old)

Wild animals

n/a

Field-collected samples

mice were obtained from the Animal Centre of Yangzhou University (Yangzhou, China), and the permission number is SCXK(su)2017-007. Mice were maintained under a 12 h light/12 h dark cycle in specific pathogen-free (SPF) conditions at 22-24 oC. All mice were fed normal chow diets (For C57BL/6J and BALB/C mice, Rodent diet, 1010041, Shoobree, Xietong Organism, Jiangsu, China; For BABL/C-nu/nu mice, Rodent diet, 1010019, Shoobree, Xietong Organism, Jiangsu, China) and water ad libitum and were treated in strict accordance with the Nanjing University guidelines (Permit NO. 2011-039) and the ARRIVE guidelines (McGrath et al., 2015).

Ethics oversight	Nanjing university has approved the study protocol, and all experiments were treated in strict accordance with the Nanjing University guidelines (Permit NO. 2011-039) and the ARRIVE guidelines (McGrath et al., 2015).

Note that full information on the approval of the study protocol must also be provided in the manuscript.

Flow Cytometry

Plots

Confirm that:

☒ The axis labels state the marker and fluorochrome used (e.g. CD4-FITC).

☒ The axis scales are clearly visible. Include numbers along axes only for bottom left plot of group (a 'group' is an analysis of identical markers).

☒ All plots are contour plots with outliers or pseudocolor plots.

☒ A numerical value for number of cells or percentage (with statistics) is provided.

Methodology

Sample preparation	Cell suspensions from the blood, spleen, or tumour tissues were filtered through Nylon cell strainers (70 µM, Falcon, USA), and red blood cells (RBCs) were lysed. After cells were washed with PBS containing 1% BSA, cells were blocked with 1% BSA at 4 °C for 30 min, according to product manuals.
Instrument	BD FACSCalibur or Attune NxT
Software	Flowjo software (Tree Star, San Diego, CA, USA)
Cell population abundance	n/a
Gating strategy	All samples were stained with antibodies or isotype control. All samples were gated on FSC-H/SSC-H to remove debris, 7-AAD and Fixability viability Dye 520 to determine live/ dead cells and then FITC-H, PE-H, APC-H and PE/Cy7-H were used to differentiate positive or negative populations. Cells stained with isotype control was used to define negative population.

☒ Tick this box to confirm that a figure exemplifying the gating strategy is provided in the Supplementary Information.
